# Topics of Public Interest

**Published:** 1914-10

**Authors:** 


### TOPICS OF PUBLIC INTEREST.

---

**Statistics of Medical Education.** Every physician should read the Educational number of the Journal of the A. M. A., August 22, 1914. Excepting for an insignificant increase in students (not graduates) at eclectic colleges, it shows a steady diminution of students, graduates and colleges and of special problems. For the year just closed, there were 16,502 medical students, 3,594 graduates, of which all were from non-sectarian colleges except 154 from homeopathic and 70 from eclectic colleges, 101 colleges, including 10 homeopathic and 4 eclectic. There were only 25 women graduates for the whole country although there were 54 co-educational schools and the prejudice against women in medicine has practically disappeared, so far as any active manifestation of it is concerned. 6 colleges give preliminary courses only and do not confer degrees. The four-year course is universal, with a minimum of 29 weeks, excepting in two instances and up to 36 weeks as a maximum. 68 colleges are rated as Class A, 19 as Class B and 14 as Class C. 74% of the graduates are from Class A colleges, 19% from Class B and 7% from Class C. At the present rate of decrease, there will be about 3500 graduates in 1915. According to Polk, there are now 132,770 physicians in the continental U. S., whose death rate in the next year will amount to about 2650 at an average of 2% per annum for the ages represented. The increase of 850 will correspond to an increase in population of about 1.9 million.

---

**Fee Bill Under Workmen's Compensation Act.** The Committee of the Medical Society of the State of N. Y. has presented a preliminary fee bill which, to put matters mildly, is not extortionate. Considered as a personal obligation of a poor workingman—not a skilled laborer at current rates of wages—to a physician of small experience or low grade of skill, the rates may be considered as fair. As an insurance, distributing the risk and obviating a financial emergency to an individual, and on the hypothesis that competent surgeons are to be employed, the rates give the impression of being inadequate. The highest fee for operation and all subsequent treatment is $75., for amputation of the hip, fixation of kidney, laminectomy and fracture of the acetabulum with wiring or plating. The ordinary run of fractures, dislocations and other operations are listed at $10-$25 for full treatment. The charge for an office call is $1.00, for a call at home $1.50. Itemized charges are not to exceed the flat rate.

---

**Decision as to Pollution of Water.** In 1911, suit was brought by the "People" in behalf of Auburn, against the trustees of the Union Free School of Moravia, for allowing sewage from about 300 pupils to enter Owasco Lake. After a long legal fight and disagreement of jury, the case has finally been decided in favor of the People. Two counts of the indictment were, however, dismissed: "Offending Public Decency" and "Annoying, injuring and endangering the comfort, repose, health or safety of a considerable number of persons" but the count of "Rendering a considerable number of persons insecure in life or the use of property" was sustained. Under the circumstances, to the non-legal mind, the three counts appear substantially identical. From the sanitary standpoint, the main item of the defense was that the lake, ten miles long, had no appreciable current but this was met with observations of Mr. Ackerman, engineer of the Auburn water board showing that the surface current, with prevailing winds from the south, would carry infectious matter to Auburn in two or three days. In 1908, an epidemic of 34 cases of typhoid in two months, occurred in Auburn, which was apparently traceable only to the above sewage infection.

---

**Examination for Assistant Surgeon, U. S. Public Health** Service will be held October 19, in several cities. Candidates should apply promptly to the Surgeon General, Washington, D. C. The salary is $2000 plus quarters or commutation at $30 per month, with opportunity for promotion. This is the best of the government services, as most of the assignments are to large cities, there is an abundance of clinical work, and one's superior officers are physicians.

---

**Fraudulent Agent Victimizing Physicians.** The Current Literature Publishing Co. warns against an agent, 21 years old, six feet tall, weighing 180 pounds, black hair, very dark and large eyes, rather pale, deep falsetto voice. $25 reward for his apprehension. Wire Periodical Publishers' Co., 156 Fifth Avenue, N. Y. City for instructions.

---

"**White Slavery.**" The Burning Bush, a good, old-fashioned, orthodox religious journal that believes in a hyperthermic hereafter, quotes from an unnamed exchange that this country spends three billion dollars a year on white slavery. We need not state that there is no possibility of such numbers of women literally held in forcible restraint or of such earning power on the part of the individual slave. Obviously, white slavery is a euphemism for prostitution. Let us see how the statement works out for prostitution. Our population of a hundred million is a trifle over 50% male. Slightly less than 25% of it is included between the age limits of 20 and 34. Considering age and sex alone, there are only about 12 million persons in the country available for prostitution. We don't know what proportion of women are prostitutes or what the average prostitute earns in a year, but, according to the source of this statement, there are either a million prostitutes at $3000 earning capacity a year or fewer at a higher earning capacity or more at a lower one. We do not believe that the average prostitute earns anything like this amount or that anywhere near one woman in twelve, of appropriate age, is a prostitute. Let us consider the matter from the male standpoint. There are scarcely 30 million males in the country, for all ages beyond adolescence. This number includes both married and single, of all grades of virility and morality. Without entering into disgusting details of potency and prices, but throwing ideals of decency to the winds, it is still the wildest absurdity to claim that the average man, throughout his entire adult life, averages $100 a year for prostitution. We think our contemporary has been caught by that kind of exaggeration which some persons consider justifiable to bolster up any reform movement.

---

**The Alvarenga Prize** for 1914 has been awarded to Dr. Herman B. Sheffield of New York, for an essay on Idiocy and Allied Mental Deficiencies in Infancy and Early Childhood.

---

**Teaching Fellowships in the Medical Department of the University of Minnesota.** The Medical School of the University of Minnesota has adopted the principle of teaching fellowships in the clinical departments, with the end in view of providing well-trained, full-time assistants and research workers and at the same time giving a basis for graduate instruction in the various specialties. It is arranged that the fellowships be in three grades, viz., first year, $500; second year, $750; third year, $1,000.

To be eligible to a first-year fellowship, a candidate, as a general rule, must have received his M. D. degree from an acceptable school and have served one year as interne in a good hospital. The fellows appointed under this system will give their entire time to study, research and such assisting in clinics as they may be prepared for. A course of study will be laid out for each fellow, adapted to prepare him for the specialty chosen by him. This course will include work in the laboratory branches, dispensary service, hospital service and investigation. It is probable that the course (of two or three years) will lead to a degree properly recognizing the specialty in which the candidate has worked. Arrangements may be made whereby these fellows can spend one year at the Mayo Clinic if desired and count the same toward the advanced degree.

In order to inaugurate the system the Board of Regents of the University has authorized the following teaching fellowships for the next school year: one each in Medicine, in Surgery, in Obstetrics and Gynecology and in Eye, Ear, Nose and Throat; each of $500. There is also provision for one $500 fellowship and one $1,000 fellowship in Mental and Nervous Diseases, or in lieu of these a $1,500 instructorship. Applications for these positions may be sent to the Dean, University of Minnesota Medical School, Minneapolis.—St. Paul Med. Jour.

---

**The War.** There are only three countries in the world in which there is the combination of national strength and monarchic power adequate to start such a war as is at present in progress—Germany, Russia and Japan. With general popular education, and the development of a demand for personal comfort and wealth, the expense of war renders an offensive war almost impossible. Emperor William is freely quoted as having prophecied that his own dynasty would soon come to an end although it might have continued for several generations in the rather humiliating form in which royalty persists in England. It was, therefore, a case of "now or never."

What has surprised us is the almost entire absence of a sense of loyalty to the mother or ancestral country in America. Native born and even naturalized persons as a rule regard the war critically and without the spirit of "My country, right or wrong." From the practical standpoint, it is well that our citizens feel themselves solely American yet it is almost a pity that so little of the filial spirit of patriotism is manifest. In 1600, Poland was the largest, strongest and most cultured country of Europe. There is hope of its reestablishment. Peace in Europe can, apparently, only be secure, when political unity is along the lines of common race and language, both because, when those conditions are fulfilled, internal dissensions will cease and because there will also be a fairly equal balance of power sufficient to prevent wars of conquest.

The magnitude of the present struggle eclipses everything else in the history of the world. While one can imagine quantitatively more destructive engines of war, and, conversely, quantitatively more resistant fortifications, there is only one conceivable adaptation of science to warfare that has not been put into operation: A rapidly moving, subterranean vehicle of attack. We even have armored automobile batteries surpassing naval developments in efficiency up to 50 years ago. On the other hand, at the present stage of the war, it appears that modern war-ships are relatively inefficient except for purposes of mutual destruction if at all equally matched and, considering the expense and comparatively small number of men possible on a ship, it seems questionable whether, except for police duty in the sense of the exercise of authority over a relatively defenseless enemy, navies will be maintained, on the scale recently demanded. If the future progress of the war confirms this hypothesis, it will be quite in analogy with the abandonment of armor for soldiers, and reliance on the law of chance for escape, forced on military men by the development of firearms some centuries ago. We hope that the inefficiency of the modern type of war ship, relatively to its cost and carrying capacity, will be demonstrated, for such a demonstration will remove one of the largest individual items of expense of governments.

We can not but be impressed with the close interdependence of the whole civilized world. A century ago, we would just about be in receipt of the first news that there was a war in Europe. At the time of writing, the editor has had levied on him, by monarchs to whom he owes no allegiance, a monthly tax of about $20. This is a tangible evidence of the wonderful progress which has been made in civilization. This war ought to teach the U. S. the necessity of minding its own business in a double sense. In a dozen ways, we find ourselves destitute of the necessities of civilized life which we might just as well produce ouurselves but which we have left undeveloped because as a people, we are not willing to do any work except of an easy kind, at short hours and for high pay.

We had hoped to present a careful, authoritative report on the drugs and other medical supplies which we have been in the habit of importing. Such information, we have been surprised to find very difficult to secure. A few instances may be mentioned as illustrations: Physicians are largely dependent on Russia for mineral oil, simply because American oil is not at present, refined to a sufficient degree of purity. We import menthol from Japan, with an abundance of peppermint growing all about us. Hydrastis has risen to an enormous price from sheer neglect of the collection of the wild plant and of the cultivation in swampy land, otherwise nearly waste, of more raw material. The active principles can be prepared artificially but they are held at prices corresponding to the natural product under present condition of supply and demand. This general subject requires serious investigation and the adoption of practical methods first to secure the gathering of native medicinal plants, many of which are abundant, and, secondly, the cultivation of such imported plants as can be grown to physiologic efficiency in our climates and soils or else the synthetic preparation of active principles. We understand that no biologic product, of nutritive, physiologic or bacteriologic nature, depends upon the foreign market, with the exception of a few antivenoms obviously not needed in America. The ability of our country to produce inorganic drugs and chemicals should be similarly studied. So far as instruments and similar paraphernalia are concerned, any dependence on imports must be due solely to lack of inventive genius, technical skill, and ability to market at a reasonable price.

Not being a political economist, we fail to appreciate why the export of materials useful in themselves in exchange for money, is a good thing for any country able to produce its own materials. Still less do we comprehend why the shutting off of exportations should raise the price of such commodities. At the risk of appearing to enter into politics, we wish to commend heartily, the action of the President in putting his foot down on the proposition to extend and increase the income tax. While appreciating that revenues must decline so far as they depend upon importations necessarily diminished by the war, we incline strongly to the amateurish belief that additional revenues should be raised merely to make good this deficit and that the indirect expense imposed on a neutral nation by the war should be balanced by a rigorous economy in other directions.

---

**Mushrooms.** The occurrence of a number of cases of poisoning, from the confusion of edible and inedible fungi and the regularity with which this experience is repeated every fall, demands the attention of the medical profession. On the average, mushrooms contain 2.5% of protein (meaning nitrogen multiplied by 6.25) 4% of fat and 6% of carbohydrate. They yield somewhat less than 30 calories per 100 grams or about a tenth as much as dry legumes. Now, considering that the protein is not strictly proteid, they rank among the lowest of the natural foods. However, it must be remembered that even economic persons spend about five times as much for food as is strictly necessary for nutritive purposes and that eating is both a nutritive process and one of the most innocent pleasures. From the aesthetic standpoint, mushrooms are among the most delicious foods and they are largely wasted from neglect. In regard to the danger of confusing edible mushrooms with poisonous fungi, it should be clearly understood—and physicians should help dispel the popular view to the contrary—

that there is absolutely no general rule, such as free peeling, color, tarnishing of silver, etc., which will distinguish the two kinds. There is no sharp distinction between mushrooms and toad stools. There are a good many kinds of edible fungi and a good many poisonous ones. The only guide to selection is either a thorough knowledge of this part of cryptogamic botany or a practical acquaintance with individual species. The ordinary mushroom hunter knows one kind of edible mushrooms and passes by several others, quite as edible. The inexpert confuses identity of appearance or blindly follows some such rule as has been quoted and death occurs.

In the case of a family, three members of which were poisoned in Buffalo, last month, the least affected member of the family went for medical aid, was arrested as insane and locked up in a cell. The groans of the other two members attracted the attention of neighbors, and telephonic reports to a police station resulted in the transfer of the supposedly insane person to a hospital. With all charity to policemen, forced to act promptly in the interest of the community, and without technical diagnostic skill, we feel that some members of the force are a little too much impressed with their own authority and somewhat too arbitrary in dealing with the public who hire them. Yet most policemen are kindly and tactful and show wonderful judgment in the discharge of their difficult duties.

---

**Liability Insurance in Massachusetts.** The Supreme Court has ruled that employers who carry liability insurance must furnish medical attendance for a period of two weeks, in case of injury to an employee.

---

**Wassermann Test.** Owing to what one of the newspapers naively terms "the popularity of the test," the Buffalo Department of Health will, from October 1 to May 1, make these tests twice a week, on Tuesdays and Fridays.

---

**Buffalo Nurses Volunteer for War.** The following nurses sailed from New York early in September: Miss Edna Reese of the Homeopathic hospital, Miss Virginia Rau of the German hospital, Miss Elizabeth Welsch, Miss Margaret Strycker and Miss Margaret Hennessey of the Woman's hospital.

---

**The Cartilage Co.** of Rochester has been convicted of using the mails to defraud, by advertising to increase stature. This reminds us of a practical problem in therapeutics: Can any one suggest a method, of course aside from the use of anodynes and extension apparatus, to relieve the pain due to pressure on nerve roots, from senile absorption of the intervertebral discs?

**Liability for X-ray Lesions.** The U. S. Supreme Court has ruled that the mere occurrence of an X-ray burn is not evidence of criminal negligence and that a Roentgenologist to whom a case is referred for examination or treatment by a competent physician is not personally responsible for making an examination to determine the suitability of the case for such examination or treatment. This leads to the inquiry whether anyone can determine, in advance, the individual degree of susceptibility of resistance to X-rays. We have recently heard of a case subjected to a hundred X-ray examinations in a brief space of time. Is such extensive application of the rays ever justifiable?

